# Chromatic processing and receptive-field structure in neurons of the anterior optic tract of the honeybee brain

**DOI:** 10.1371/journal.pone.0310282

**Published:** 2024-09-12

**Authors:** Theo Mota, Benjamin Paffhausen, Randolf Menzel

**Affiliations:** 1 Institute of Biology, Neurobiology, Freie Universität Berlin, Berlin, Germany; 2 Department of Physiology and Biophysics, Institute of Biological Sciences, Federal University of Minas Gerais, Belo Horizonte, Brazil; University of Nebraska Medical Center College of Medicine, UNITED STATES OF AMERICA

## Abstract

Color vision in honeybees is a well-documented perceptual phenomenon including multiple behavioral tests of trichromaticity and color opponency. Data on the combined color/space properties of high order visual neurons in the bee brain is however limited. Here we fill this gap by analyzing the activity of neurons in the anterior optic tract (AOT), a high order brain region suggested to be involved in chromatic processing. The spectral response properties of 72 units were measured using UV, blue and green light stimuli presented in 266 positions of the visual field. The majority of these units comprise combined chromatic-spatial processing properties. We found eight different neuron categories in terms of their spectral, spatial and temporal response properties. Color-opponent neurons, the most abundant neural category in the AOT, present large receptive fields and activity patterns that were typically opponent between UV and blue or green, particularly during the on-tonic response phase. Receptive field shapes and activity patterns of these color processing neurons are more similar between blue and green, than between UV and blue or green. We also identified intricate spatial antagonism and double spectral opponency in some receptive fields of color-opponent units. Stimulation protocols with different color combinations applied to 21 AOT units allowed us to uncover additional levels of spectral antagonism and hidden inhibitory inputs, even in some units that were initially classified as broad-band neurons based in their responses to single spectral lights. The results are discussed in the context of floral color discrimination and celestial spectral gradients.

## Introduction

Color vision, the ability to distinguish objects based on hue differences only, is a common property across multiple animal species [1,2]. The neural substrate of recognizing color patterns has been traced to combined chromatic-spatial properties of visual neurons typically organized with double opponent properties [3]. Although multiple studies document the ability of color pattern discrimination in invertebrates, particularly insects, rather little is known about the neural mechanisms of combined chromatic and spatial vision. This is particularly surprising for the honeybee since multiple psychophysical studies document combined chromatic and spatial discrimination. Honeybees use color vision in at least two behavioral contexts, flower visitation and sun compass related navigation. Angiosperm plants evolved colors and patterns as advertisement for pollinators. Hymenopteran pollinators share similar wavelength sensitivity peaks of their photoreceptors indicating a conserved pattern at the input stage of their color vision system [4–6]. Bees have an excellent trichromatic color vision system able to discriminate a large number of colors because of the equidistant position of photoreceptor sensitivity peaks across the whole visual range from 300 to 680 nm and optimal overlap of their spectral sensitivity functions with half band width of 100 nm (for review: [7,8]). Although there is no evidence for a close co-evolutionary relationship between the bee’s set of photoreceptors and flower colors, it appears that evolution of floral colors lead to a diversification on the plant side sufficient for such a perfect color vision system to operate effectively [9]. Color vision in bees may also play a role in using the celestial compass [10,11].

Multiple perceptual phenomena have been studied in color vision of bees including illusory colors in moving patterns [12], an achromatic interval at low light intensities [13], the spatial resolution of color vision versus achromatic vision [14], additive color mixing phenomena [15], spectral discrimination [16], color generalization [17], spontaneous color choice [18], color learning [19], color constancy [20,21], the Bezold-Brücke color shift [22]. The ease with which bees can be trained to a food source allowed to collect a large range of color discrimination data for constructing a two dimensional perceptual space of colored stimuli [23,24].

The trichromatic receptor input was already documented in early times of intracellular electrophysiology [25] and then combined with intracellular marking techniques [26]. Although molecular analyses uncovered more complexity in the distribution of spectral receptor types across the compound eye [27] the triplet of receptors with λ_max_ at 340, 440 and 540 nm appeared to capture best the input to the neural color coding system and the perceptual space as quantified in a Maxwell triangle [6] and a two dimensional color opponent system [23]. Color opponent neurons were recorded in the visual ganglia [28], and their chromatic properties mirrored the two opponent dimensions defined by multidimensional scaling methods of behavioral discrimination data [24]. The loci of flower colors in a receptor based Maxwell triangle [6,29] and in the color opponent space [30] allowed to address the question of mutual evolutionary relation between flower colors and the color vision system of flying hymenopteran pollinators [5]. It was found that the color vision system of flying Hymenoptera acting as pollinators is optimal and floral colors do not utilize fully their potential perceptual space.

It remains an open question how spatial distributions of chromatic contrast are processed by color-sensitive neurons. Receptive field sizes of many color-coding neurons recorded so far exceed the behaviorally determined spatial resolution of the chromatic system for flower-like objects and patterns [28,31–34]. For example, tonic color-opponent neurons in the proximal lobula and in medulla-extrinsic interneurons that project to the protocerebrum via the posterior optic commissure have diverse and complex chromatic-spatial properties including opponency between single or combined chromatic receptor signals. Furthermore, neurons were found with double spectral opponency and different inputs from the two eyes [28,31,33–38].

Whereas the receptive fields of color-opponent neurons recorded in the proximal medulla were rather homogeneous, lobula color-sensitive neurons showed variations in responses across locations in the receptive field with rather complex features, contrasting color coding midget ganglion cells in primates that are characterized by a spatial center-surround organization of receptive fields [39]. Furthermore, the receptive fields of color-sensitive neurons vary in size but tend to be rather large, usually above 30° visual angle [28,31]. These physiological properties correspond to the wide branching patterns of the respective neurons both in honeybees [31], and in bumble bees [34]. Thus the data on receptive field structure in color coding neurons is rather limited and come predominantly from neurons in the peripheral visual ganglia medulla and lobula.

Here we aim to fill this gap by extracellular multi-unit recordings from neurons of the prominent anterior optic tract (AOT), which provides input from the medulla and lobula to the anterior optic tubercle (AOTU), a higher order visual structure in the anterior lateral bee brain [40,41]. Neurons of this brain region have been found to be more strongly involved in color coding than the posterior visual stream that appears to deal more with movement coding [42]. Also, calcium-imaging of AOTU interneurons revealed spatiotemporal color coding and color opponency phenomena in this brain region [43]. The AOTU of bees appears to be involved also in the analysis of polarized light and possibly in sky compass processing, a condition that might relate to the spatial-chromatic contrast between the sun and the blue sky mentioned above [41,43–46].

The spatial and chromatic properties of AOT neurons are so far unknown in bees. We addressed the question of combined chromatic-spatial properties in honeybees by stimulating the right eye with three spectral stimuli (λ max at 360 nm, 440 nm, 520 nm) presented at 266 positions. We also applied stimulus protocols with overlapping spectral stimuli with the aim of uncovering spectral antagonism and hidden inhibitory inputs. We found that AOT neurons varied in terms of their receptive field structures and temporal as well as chromatic response properties. Both large receptive fields and spectral opponency were predominant patterns. We also identified intricate spatial antagonism and double spectral opponency in receptive fields of some color-opponent units. Our data are discussed in light of the putative role of the honeybee AOTU in color vision and navigation.

## Materials and methods

### Honeybee preparation

**Fig 1:**
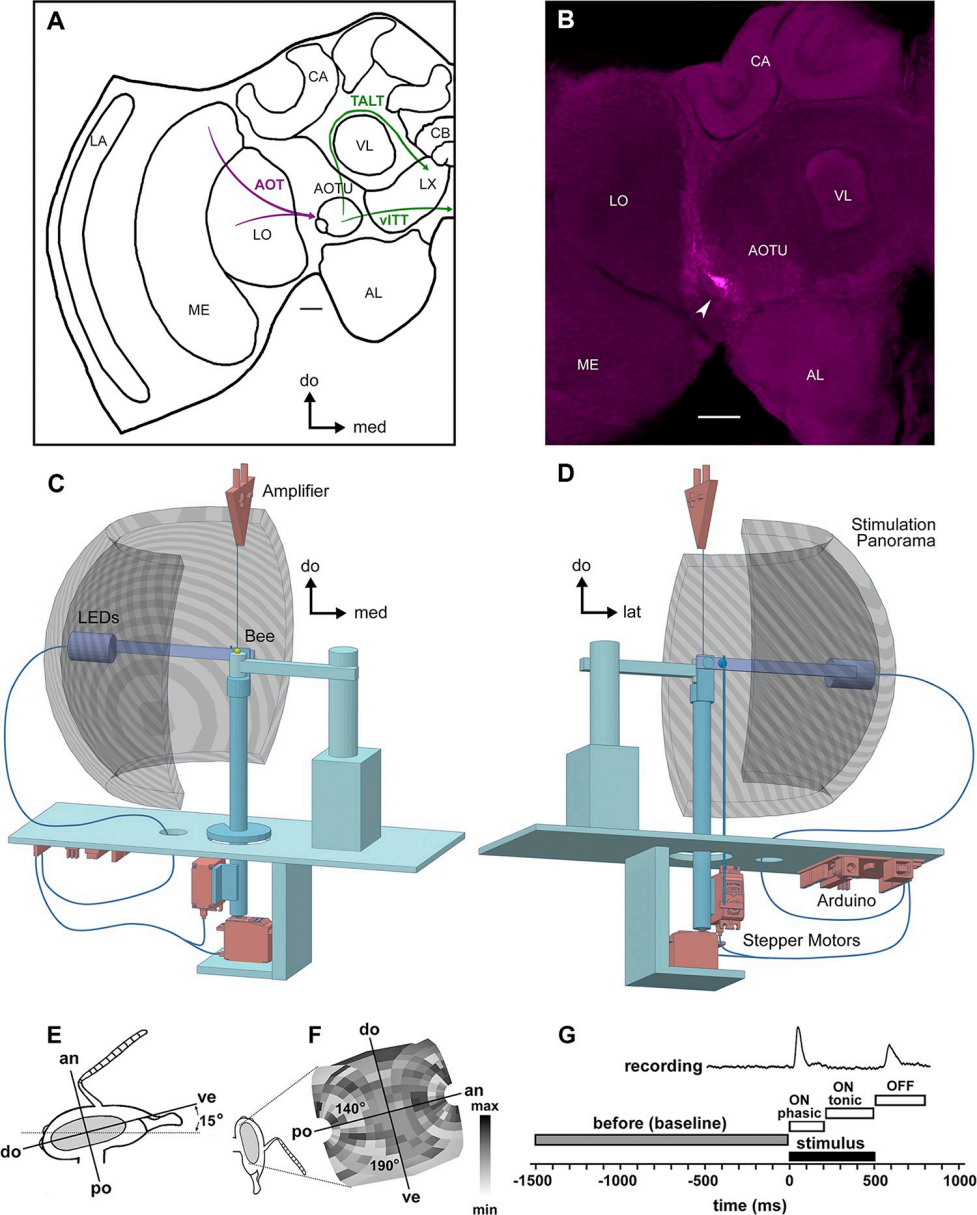
Extracellular multi-unit recording in the anterior optic tract (AOT) of the honeybee brain. **A**) Scheme of the bee brain showing the location and the main input and output tracts involving the anterior optic tubercle (AOTU). The AOTU is divided into a major unit and a small lateral ensemble of units. It receives input from the medulla (ME) and lobula (LO) via the prominent AOT (in magenta), which was the target of our neural recordings. Output (in green) to the contralateral AOTU and the lateral complex (LX) is provided by the ventral intertubercle tract (vITT) and the tubercle accessory-lobe tract (TALT), respectively. **B**) Example of *micro-ruby* fluorescent staining revealing the place where the electrodes were inserted (arrow). Electrodes were placed in the anterior protocerebral surface at the level of the AOT, thus lateral to the AOTU input region. LA: Lamina; AL: Antennal lobe; CA: Calix of the mushroom body; CB: Central body; scale bars = 50 μm. **C-D**) Elements of the stimulation and recording set-ups are presented in two different perspectives. **C**) Two electrodes connected to an amplifier were inserted into the brain of an immobilized bee. The arm pivoting around the bees head was equipped with 3 LEDs (wavelengths: 360 nm, 440 nm, 520 nm) behind a diffuser. **D**) Two stepper motors controlled the movements of the arms within the semispherical stimulation panorama. An Arduino microcontroller controlled the motors and lightening of the LEDs. One motor controls the movement of the LEDs horizontally (left to right aligns with ventral to dorsal) over a section of 190°. The other motor moves the arm vertically (up to down aligns with anterior to posterior) over a section of 140°. The voltage of the LEDs and the PWM (pulse width modulation) signals were picked up by the same analog to digital converter that recorded the electrophysiological data. The coordinates of the arms (in 266 positions) and the LEDs´ activation were randomized. **E)** Lateral view of the bee head and right eye during recording. As described by Seidl & Kaiser [50], the middle axis separating the bee eye in its anterior and posterior halves has an inclination angle of 15° in relation to the horizontal plane in which the bee head lays in the holder. **F**) Representative example of the receptive field of a recorded neuron, calculated considering the bee eye inclination and curvature [50] and the Cassini projection of the 266 positions in which visual stimuli were presented (see section ‘*receptive-field projection*’). The grayscale represents the recorded spike rate in each coordinate from minimum to maximum values. **G**) Temporal basis of computing spike rate changes for receptive field analyses. To compute the spike rate changes of the ON-phasic, ON-tonic and OFF response the average spike rate of the before time window (1500 ms before stimulus onset until 10 ms before stimulus onset) was subtracted from the average of the respective time window (ON-phasic: 10 ms—200 ms; ON-tonic: 200 ms—450 ms; OFF: 510 ms—800 ms). Anatomical axes are presented in relation to the bee right eye: do = dorsal, ve = ventral, an = anterior, po = posterior, med = medial, lat = lateral.

Forager honeybees (*Apis mellifera*) were caught at the entrance of a hive, anesthetized in glass vials by putting them on crushed ice, and transferred into a bee holder with a support for the neck. The back of the bee`s thorax and abdomen was secured by fabric tape, and the head capsule was mechanically connected to the neck plate by dental wax. When melting the dental wax around the head capsule the right eye was left free. The antennae were immobilized by n-Eicosane (Sigma-Aldrich, MO, USA) and attached to the front of the head capsule. The animal’s head capsule was opened under stereomicroscope vision, and the tracheae and glands were moved to the side to reach the anterior optic tubercle (AOTU). A custom made electrode bundle of 2 copper wires (insulated, 15 μm diameter, Elektrisola, Reichshof-Eckenhagen, Germany) served as inputs and a silver wire (bare, 25 μm, Advent Research Materials, Oxford, UK) as ground for extracellular recordings. The two input channels were gold plated [47]. Afterwards, the tips of the copper wires were covered by a very small amount of micro-Ruby fluorescent dextran (tetramethylrhodamine and biotin, 3000 MW, Invitrogen) to allow further anatomical appraisal of the electrode position using confocal microscopy. Using a micromanipulator, we inserted the pair of copper electrodes in the anterior surface of the lateral protocerebrum, at the level in which the anterior optic tract enters the AOTU (Fig 1A and 1B). The AOT is a prominent tract that provides input from the medulla and lobula to the AOTU [41]. This tract also contains some large-field neurons connecting the lobula and the AOTU of the two brain hemispheres through the ventral intertubercle tract (vITT; Fig 1A). After the electrodes were inserted into the AOT (Fig 1B) and stable spike trains were observed, the head capsule was closed by non-toxic 2-component silicone (Kwik Sil, World Precision Instruments, FL, USA). When the silicone was cured, the stimulus protocol started. While a total of 148 bees was submitted to this experimental procedure, reliable single-unit activity was obtained in 72 neurons recorded from 46 bees.

### Stimulus apparatus

The stimulating apparatus (Fig 1C and 1D) consisted of an arm movable in two axes with its pivot point at the bee’s right eye. The arm was swiveled around the bee’s eye by two stepper motors, with freedom of movement horizontally of 190° and vertically of 140° (Fig 1D). The bee-far end of the arm was equipped with three LEDs (360 nm, 440 nm, 520 nm) behind a UV transmitting diffuser (diameter 15 mm, 10° opening angle for the bee) pointing toward the bee (Fig 1C). The absolute irradiance of each spectral light was adjusted at the level of the bee eye to an equivalent value of 4.5 x E^-3^ μW cm^-2^ and monitored by means of a spectroradiometer (UV-VIS S2000 coupled to DT-1000, Ocean Optics) connected to a cosine-corrected irradiance probe (CC-3-UV, Ocean Optics). These UV, blue, and green stimuli calibrated to the same photon flux allowed maximal excitation of the S, M, or L photoreceptor types of bees, respectively [5,26]. The motors and LEDs were controlled by an Arduino microcontroller (Fig 1D; Arduino UNO R3, Arduino, Somerville, MA, USA). The PWM (pulse width modulation) signals of the motors as well as the voltage driving the LEDs were connected to inputs of an analog digital converter (1401micro, Cambridge Electronics Devices, Cambridge, UK) that recorded the electrophysiological data. A PC saved the electrophysiological data and the states of the stimulation apparatus.

### Receptive field protocol

The goal of the stimulus protocol was to illuminate every 10° raster point in the near half sphere (190° [dorsal—ventral] x 140° [posterior—anterior]) once for every spectra stimulus (Fig 1E and 1F). The coordinates (Fig 1F) and LED stimuli were chosen in a pseudo random sequence under the control of the Arduino. Thus all experimental bees were subject to the same random pattern of stimulations. The motors moved the arm to the appropriate position, paused for 10 s and then presented the LED stimuli for 500 ms. The cycle was repeated with the next coordinates and LED stimuli according to the pseudorandom table of the Arduino. We recorded the activity of 72 neurons from 46 bees using this protocol.

### Color opponency protocol

We defined six coordinates (anterior-dorsal, anterior-ventral, posterior-dorsal, posterior-ventral, medial-dorsal and medial-ventral) within the bee’s visual field and applied 10° stimulation with distinct chromatic mixtures three times per coordinate. A mixture of all three LEDs (bee’s white) was presented for 500 ms. Also, a background color (UV, blue or green) was presented for 700 ms, and 100 ms after its onset a second color was switched on for 500 ms. Thus seven types of stimuli were presented in each of the six coordinates: bee’s white, uv-blue, uv-green, blue-uv, blue-green, green-uv, green-blue. Again, the order of coordinates and visual stimuli was pseudorandom. We recorded the activity of 21 independent neurons from 21 bees using this protocol. All the 21 bees tested with this color opponency protocol were first submitted to the receptive field protocol previously described.

### Anatomical appraisal of the electrode position

Tracer-filled brains were dissected out after successful electrophysiological recordings. Brains were fixed in phosphate-buffered paraformaldehyde solution (4%; pH 6.8) for at least 24 h, dehydrated in ascending concentrations of ethanol, and cleared in methyl salicylate (Sigma-Aldrich) for 24 h. Images of whole mounts were taken with a Leica TCS SP8 confocal microscope, 8-bit, (1,024 x 1,024 pixels) with a 10X / 0.3na HC PL FLUOTAR WD 11 mm objective. Projections of the confocal stacks containing *micro-Ruby* (Invitrogen) dextran staining (Fig 1B), as well as brightness and contrast adjustments, were achieved using the open source imaging software FIJI [48].

### Data analysis

The data consisted of two raw spike train channels, the voltage of the 3 LEDs and the PWM signal controlling the motors. The spikes were sorted into single spike templates by the semi-automatic sorting process of spike2 (version 7, Cambridge Electronics Design, Cambridge, UK). Briefly, to detect and verify single unit activity and differentiate them from multi-unit activities we performed the following steps. First, the whole trace was visually inspected to search for a disturbed baseline which would indicate movement of the electrode. If the recording had a stable baseline, we proceeded. A threshold of minimum spike amplitude was applied to sort noise from neural spikes. The sorting package of spike2 software clustered spikes of equivalent shape and presented these clusters into a principal component analysis (PCA). If a cluster was completely separated from any other group and presented typical neuronal spike shapes with stable amplitude between spikes, we assumed this cluster contained single unit activity. If the spikes were of characteristic shape but their amplitude and specific shape varied within the detected cluster, we assumed a multi-unit activity originated by more than one neuronal source. Single unit activity rates are eminent due to the refractory period between their spikes. We thus confirmed single-unit activity by checking minimal inter-spike intervals in each cluster of interest.

The data were further analyzed by custom written MatLab (R2019a, MathWorks, MA, USA) scripts. Evoked activities produced by each visual stimulus in the 266 positions of stimulation were visualized in raster plots and average peristimulus time histograms (PSTH). Spike frequency changes were computed by subtracting the average baseline activity 200 ms before the stimulation from the average spike rate of the following response phases after stimulus onset: ON-phasic: 10 ms—200 ms; ON-tonic: 200 ms—450 ms; OFF: 510 ms—800 ms. These time-windows were defined based on the average PSTHs of all sorted single units, which revealed potential changes of spike frequency in these three response phases. In each of these three phases, we considered as excitatory or inhibitory responses, spike rate changes above or below 2 standard deviations (SD) from the average baseline rate, respectively.

After the analyses described above, each unit was classified as broad-band, narrow-band or color-opponent (Table 1) based on their average PSTHs (responses in 266 positions) to the three spectral stimuli (UV, blue and green). Broad-band neurons present similar responses to all colors, narrow-band neurons respond to only one or two of the colors, and color-opponent neurons present different responses to individual colors [28,32].

**Table 1:** Distinct chromatic categories of neurons recorded in the honeybee AOT.

Spectral sensitivity	Response property		Number / Percentage
**Broad-band neurons**		**26 / 36%**	
UV+ / B+ / G+	ON tonic		03 / 04%
UV+ / B+ / G+	ON phasic		11 / 15%
UV+ / B+ / G+	ON/OFF phasic		12 / 17%
**Narrow-band neurons**		**12 / 17%**	
B+ / G+	ON phasic		12 / 17%
**Color-opponent neurons**		**34 / 47%**	
UV+ / B- / G-	ON phasic-tonic/ OFF phasic		13 / 18%
UV(s) / B(f) / G(f)	ON/OFF phasic		12 / 17%
UV(f) / B(s) / G(s)	ON/OFF phasic		05 / 07%
UV(on)/B(on-off)/G(on-off)	ON to UV/ ON-OFF to B or G		04 / 05%

### Receptive field projection

Representation of receptive field responses was achieved by arranging the coordinates of the 266 positions stimulating to the bee right eye (Fig 1E) in a Cassini projection (Fig 1F). A flat rectangular array would not do justice to the rather small contribution of the longitude near the poles. To account for these distortions when flattening a near half sphere into a 2D projection using the Cassini projection [49]:

$$x=\arcsin\;(\cos\;\varphi \;\sin\;\lambda )\;y=\arctan\;(\frac{\tan\;\varphi }{\cos\;\lambda })$$

The structure of the receptive field was further adjusted considering the bee eye inclination and curvature (Fig 1E and 1F) described by Seidl & Kaiser [50]. A grayscale is used to represent spike rate from minimum to maximum values (Fig 1F). Spike frequency changes in each receptive field position are presented in false color (Figs 4 and 5). Warmer colors (green-yellow-red) correspond to higher rates, colder colors (blue-purple) to lower rates (turquoise; no spike change). Light grey areas show positions in the receptive fields that were missing by either abrupt ends of recordings or, unfortunately due to light stimulation failures that could not be corrected (Figs 4 and 5). The spike frequency change was computed by subtracting the average baseline activity before the stimulation from the average spike rate of the time window of interest (Fig 1G). Spike rate changes needed to exceed 2 standard deviations (SD) from the baseline spike rate changes to be detected. Every change that was smaller is depicted in the same light blue shade as no change, and all spike rate changes above (excitation) or below 2 SD (inhibition) are depicted accordingly (Figs 4 and 5). The short and the long axis of each receptive field were estimated as the amount (in degrees) of continuous excitatory or inhibitory data points (10° each) in the anterior-posterior and dorsal-ventral axes of the Cassini projections.

### Statistical analysis

Friedman tests followed by Wilcoxon matched pairs tests were used to compare the following parameters: (i) the sizes of the short and long receptive field (RF) axes recorded for UV, blue or green (Figs 4D and 5C); (ii) overlap between excitatory and/or inhibitory RFs recorded for UV, blue or green (Figs 4E and 5D); (iii) spike rates of a unit in response to distinct spectral stimuli and chromatic combinations presented in distinct RF positions (Figs 7–9). During RF size analysis (i) performed in neurons of a same response type (Table 1), each single unit was treated as an independent variable and responses to each stimulation were treated as repeated (dependent) measurements performed in a same neuron. For calculation of the overlap between excitatory and inhibitory receptive fields (ii), each region was considered as excited or inhibited if it presented spike rate changes above or below 2SD from baseline, respectively. Bonferroni corrections were applied for multiple comparisons. Generalized linear models (GLM) followed by Tukey’s HSD post-hoc tests were developed to compare the RF sizes between different neuron types (type effect) in response to different light colors (color effect). All statistical analyses were performed in the R environment [51].

### Ethics statement

This study does not require an ethics statement, since it was performed in the honeybee. Even if research using this animal model does not require approval from an institutional animal care and usage committee, we applied classical methods of manipulation, anesthesia and euthanasia that are known to minimize stress and suffering in insects.

## Results

### Chromatic properties and receptive field structure in AOT neurons

We recorded 72 units in the anterior optic tract (AOT) of the honeybee lateral protocerebrum using UV, blue and green light stimuli presented with equal irradiance in 266 positions of the ipsilateral visual field (Fig 1). In order to characterize the chromatic and spatial properties of these high-order visual neurons we first describe the chromatic categories defined by their average responses to the three spectral stimuli and find three main groups with their particular subgroups (Table 1).

Units were classified as broad-band, narrow-band or color-opponent based on their average responses (from PSTH of 266 presentations) to the three spectral lights [28,32]. Broad-band neurons presented similar responses to all colors, narrow-band neurons respond to only blue and green, and color-opponent neurons presented different responses to distinct colors. Subgroups were identified within the same chromatic category by their temporal properties: ON or ON/OFF; phasic, tonic or phasic-tonic (see section ‘*data analysis*’). Signs + or—indicate excitatory or inhibitory responses, respectively. Slow or fast adaptations to sustained stimuli are represented by s or f, respectively.

### Broad-band neurons

**Fig 2:**
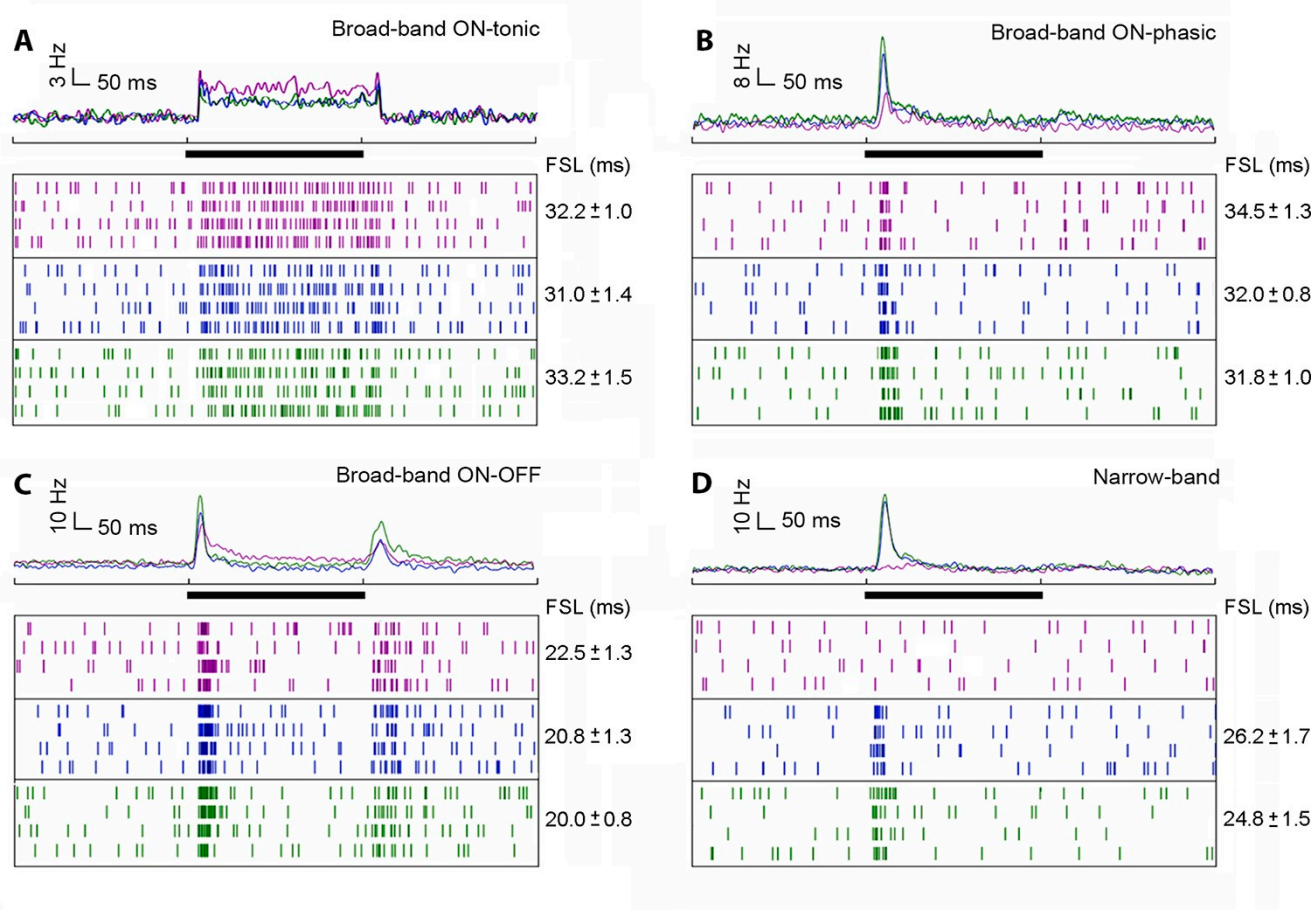
Broad-band and narrow-band units recorded in the AOT. Average peristimulus time histograms (PSTH) of 266 presentations are shown above spike raster plots across four presentations of each spectral light (UV, blue and green). **A:** Broad-band ON-tonic unit; **B:** Broad-band ON-phasic unit; **C:** Broad-band ON-OFF unit; **D:** Narrow-band unit. Each example of single unit shown in A-D come from a distinct bee. Timing of light presentation (= 500 ms) is indicated by the black bar. First spike latency (FSL; mean ± SD) in response to each stimulus is shown at the right of each respective raster plots.

Broad-band units (n = 26; Table 1) were excited by all three spectral stimuli (UV, blue and green, response type: UV+/B+ /G+). Despite this common property, these neurons were subdivided according to three distinct physiological categories: *ON-tonic neurons* responded with tonic excitation to all three stimuli (n = 03; Table 1). The excitatory response amplitude was slightly higher for UV than for blue and green lights (UV > B/G; Fig 2A). *ON-phasic neurons* had pronounced excitatory phasic responses with fast adaptation (n = 12; Table 1). Excitatory response amplitude was typically lower for UV than blue and green lights (UV < B/G; Fig 2B). *ON-OFF neurons* responded with excitatory phasic responses to spectral light onset, followed by strong excitatory off-response and slow adaptation (n = 11; Table 1). The excitatory response amplitude was typically lower for UV than blue and green lights (UV < B/G; Fig 2C).

### Narrow-band neurons

Narrow-band neurons (n = 12, Table 1) were sensitive to blue and green light, and did not respond to UV light (Fig 2D). They typically presented excitatory phasic responses with fast adaptation both to blue and green lights (Fig 2D).

### Color-opponent neurons

**Fig 3:**
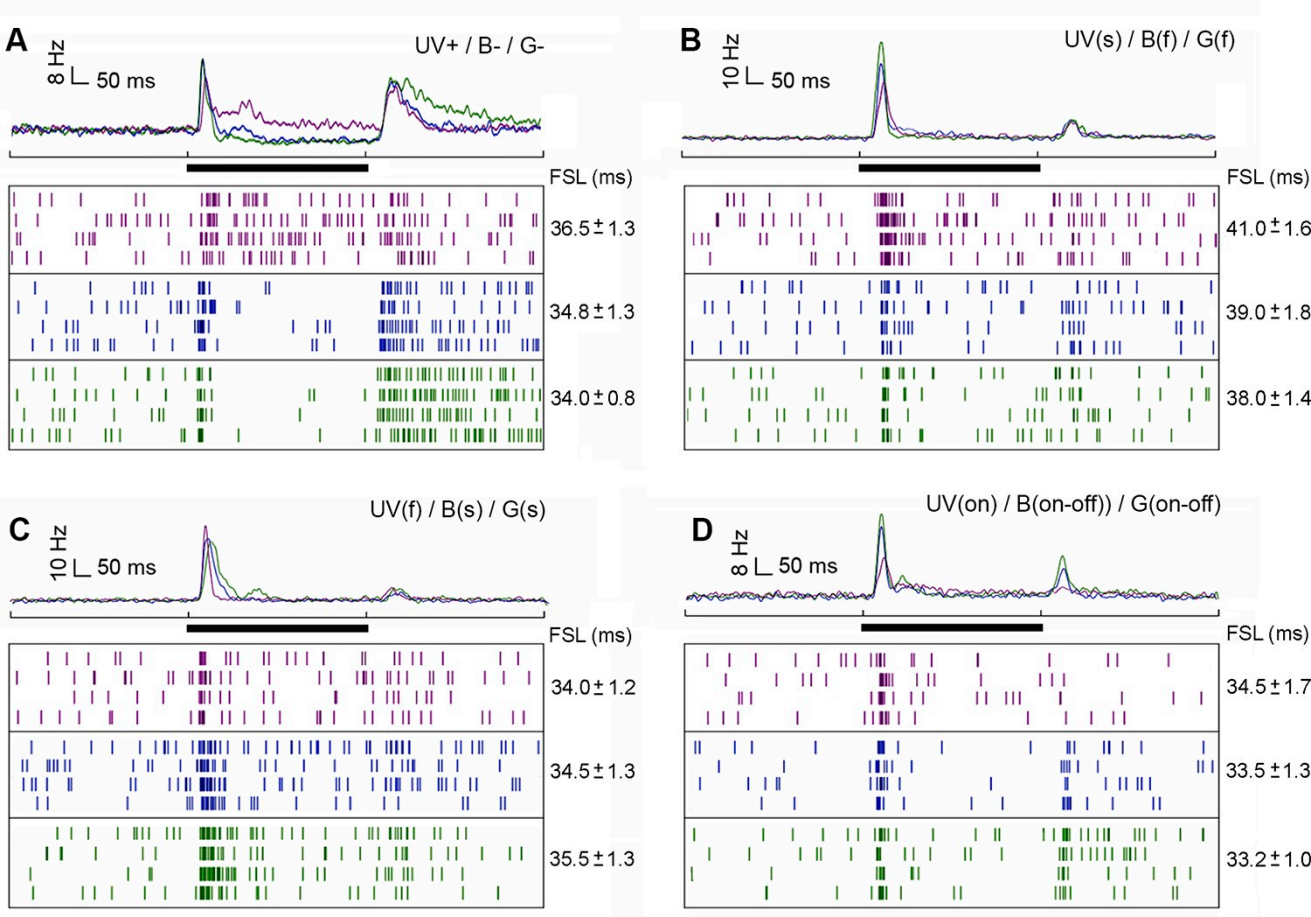
Color-opponent units recorded in the AOT. Average peristimulus time histograms (PSTH) of 266 presentations are shown above spike raster plots across four presentations of each spectral light (UV, blue and green). **A:** UV+ / B- / G- unit; **B:** UV(s)/B(f)/G(f) unit; **C:** UV(f)/B(s)/G(s) unit; **D:** UV(on) / B(on-off) / G(on-off) unit. Each example of single unit shown in A-D come from a distinct bee. Timing of light presentation (= 500 ms) is indicated by the black bar. First spike latency (FSL; mean ± SD) in response to each stimulus is shown at the right of each respective raster plots.

All color-opponent neurons (n = 34) responded to UV stimuli differently than to blue and green stimuli. Despite this typical spectral opponency (UV *versus* blue/green), these neurons were grouped into at least three distinct physiological subcategories. *UV+ / B- / G-* units had excitatory phasic on-responses to UV, blue and green (n = 13; Table 1), followed by tonic excitation to UV and tonic inhibition to blue and green stimuli (Fig 3A). They also showed strong excitatory off-responses with slow adaptation to all three spectral stimuli. In general, the on and off phasic components were higher for green and blue than for UV stimuli (UV < B/G; Fig 3A). *UV(s)/B(f)/G(f)* or *UV(f)/B(s)/G(s)* units had excitatory phasic on-responses to UV, blue and green, followed by adaptation that differed between stimuli (*s* = *slower*; *f* = *faster*; Table 1). Typically, adaptation was slower (n = 12; Fig 3B) or faster (n = 5; Fig 3C) in UV when compared to blue or green (Table 1). This color-opponent category also showed excitatory off-responses to UV, blue and green stimuli (Fig 3B and 3C). In *UV(s)/B(f)/G(f)* units (Fi. 3B), phasic on-responses were usually higher for green, followed by blue and UV stimuli (UV < B< G). Conversely, phasic on-responses in *UV(f)/B(s)/G(s)* units (Fig 3C) were higher for UV than for blue and green (UV > B/G). *UV(on) / B(on-off) / G(on-off)* units had excitatory phasic on-responses for all three spectral stimuli (n = 4; Table 1), with lower responses to UV than to blue and green (UV < B/G; Fig 3D). In this color-opponent category, neurons gave excitatory phasic off-responses to blue and green lights, but not to UV light (Fig 3D).

### Receptive field structure in distinct response phases

**Fig 4:**
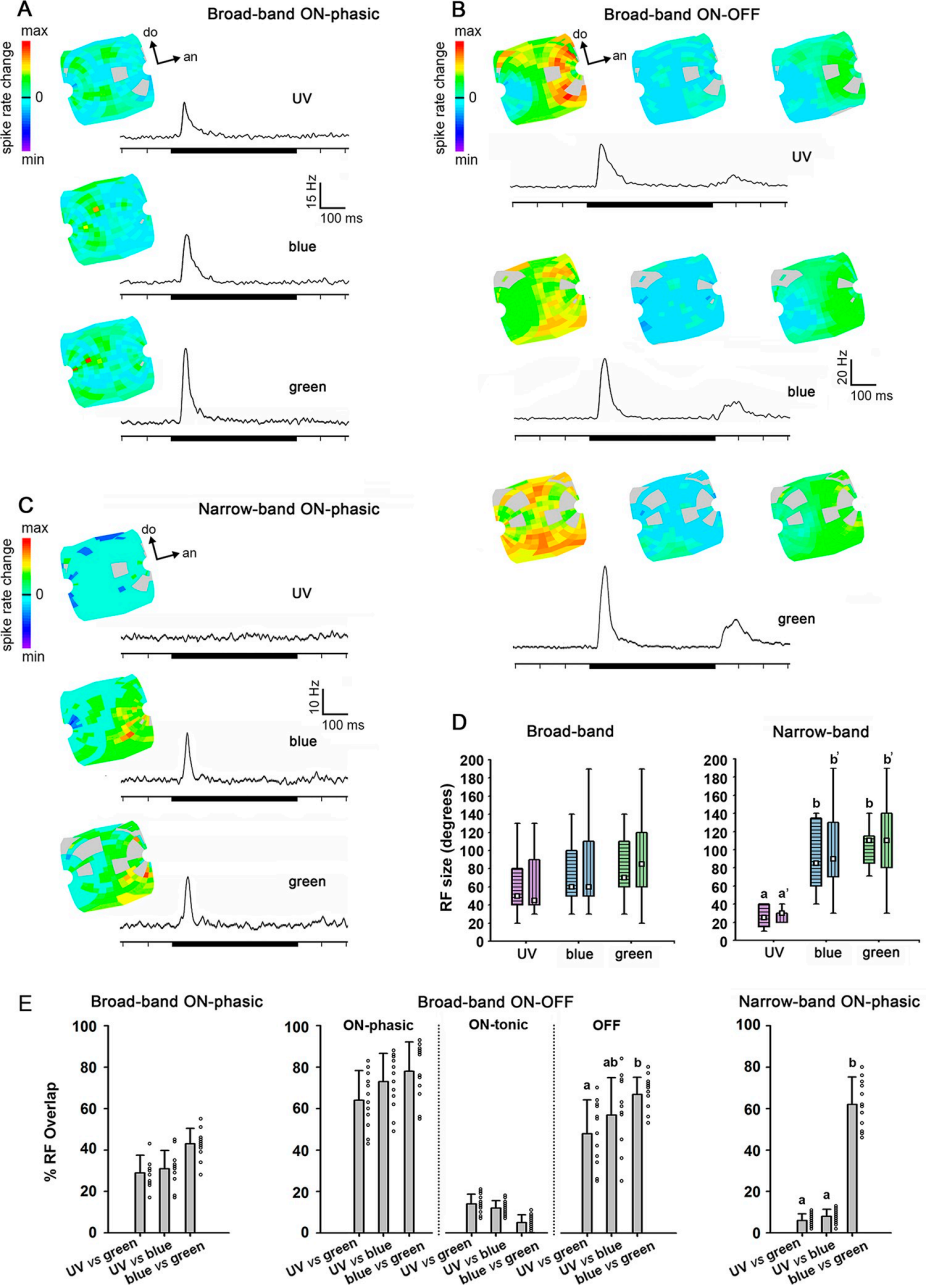
Receptive field (RF) structure of broad-band (BB) ON-phasic (A), broad-band ON-OFF (B) and narrow-band (NB) AOT neurons (C). **A** and **C**: RF activation during ON-response phase and average PSTH are shown for each spectral stimulus (UV, blue and green). Black bars indicate the stimulus presentation of 500ms. **B**: Equivalent to **A** and **C**, but RF activation is also shown during the OFF-response phase for a BB ON-OFF neuron. Each example shown in A-C is from a different bee. Light gray indicates regions of the RF where light stimuli failed or were unreliable. **D**: Average RF sizes recorded for UV, blue and green in BB (left; N = 26 units recorded in 22 bees) and NB (right; N = 12 units recorded in 12 bees) neurons. Short and long axes of the RF are indicated by horizontal and vertical stripes within boxplots, respectively. The white square inside the box shows the mean. The boundaries of the box indicate the 25th and 75th percentile. Whiskers indicate the minimum and maximum values. **E**: Overlap between RFs for each pair of spectral stimuli (UV *vs* blue; UV *vs* green, blue *vs* green) in BB ON-phasic (N = 11 units; 10 bees), BB ON-OFF (N = 12 units; 11 bees) and NB neurons (N = 12 units; 12 bees). The dots at the right of bars show individual plots of each independent unit. Different letters indicate significant differences (p < 0.05) in Wilcoxon matched-pairs tests.

**Fig 5:**
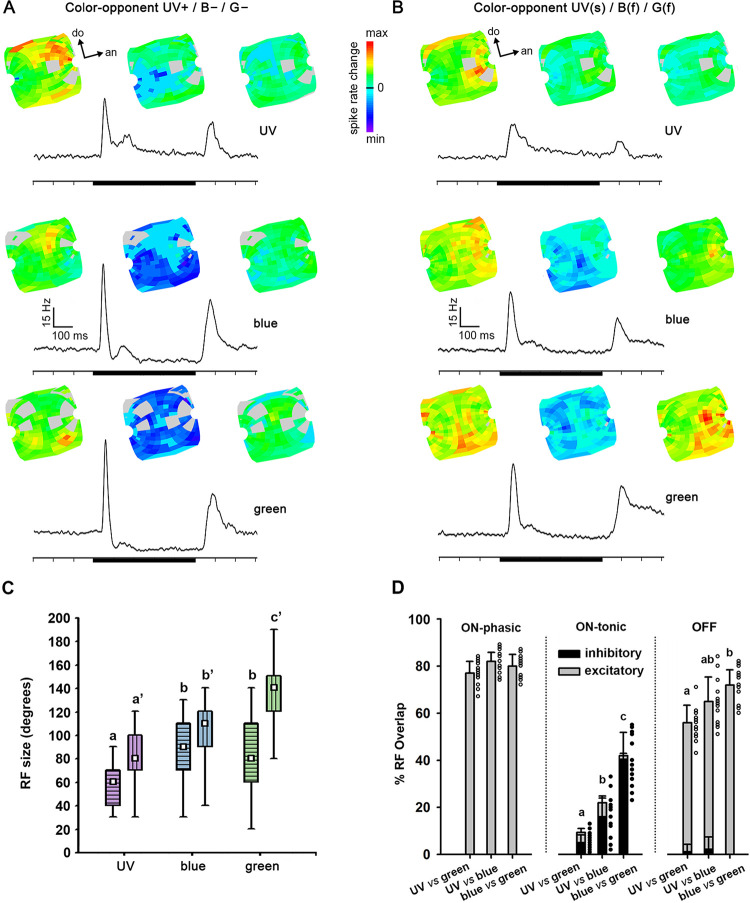
Receptive field (RF) structure of a UV+/B-/G- (A) and a UV(s)/B(f)/G(f) color-opponent AOT neuron (B). RFs expressed for excitation and/or inhibition are shown during the ON-phasic (left), the ON-tonic (middle) and the OFF (right) responses. Average PSTHs of 266 presentations are presented below the RFs for each spectral stimulus (UV, blue and green). Black bars indicate the stimulus presentation of 500ms. Examples shown in A and B were recorded in distinct bees. Light gray indicates regions of the RF where light stimulation failed or appeared to be unreliable. **C**: Average RF sizes recorded for UV, blue and green in color opponent neurons (N = 34 neurons recorded in 29 bees). Short and long axes of the RF are indicated by horizontal and vertical stripes within boxplots, respectively. The white square inside the box shows the mean. The boundaries of the box indicate the 25th and 75th percentile. Whiskers indicate the minimum and maximum values. **D:** Overlap between RFs for each pair of spectral stimuli (UV *vs* blue; UV *vs* green, blue *vs* green) in UV+/B-/G- color-opponent neurons (N = 13 neurons; 13 bees). The dots at the right of bars show individual plots of each neuron recorded. Excitatory RF overlap is represented by gray and inhibitory RF overlap by black bars and dots. Different letters indicate significant differences (p < 0.05) in Wilcoxon matched-pairs tests.

The receptive fields (RF) of units varied in size, and were rather large (Figs 4 and 5). During the phasic on-response, broad-band units (Fig 4A and 4B) and narrow-band units (Fig 4C) presented significantly larger RFs for green and blue, than for UV light (Fig 4D; Friedman ANOVA, Chi^2^ > 15.22, color effect: p < 0.001; Wilcoxon test, green *vs* UV: p < 0.001, blue *vs* UV: p < 0.01; both for long and short axes across the RF). Mean RF sizes for green and blue lights did not differ in broad-band or narrow-band units (Fig 4D; Wilcoxon test, blue *vs* green: not significant–NS, both for long and short axes). Interestingly, ON-OFF responses lead to larger RF sizes than ON-phasic (e.g. Fig 4A and 4B) or ON-tonic broad-band neuron (Table 1; GLM; type effect: F = 25.51, df = 2, p < 0.001; type x color interaction: F = 0.96, df = 4, NS).

The mean overlap between the RFs for each spectral stimulus (UV, blue, green; Fig 4A) did not differ in broad-band ON-phasic units (Fig 4E; Friedman ANOVA, Chi^2^ = 4.91, NS). In broad-band ON-OFF neurons (Fig 4B), as well, mean overlap between RFs to each pair of stimulus did not differ during ON-phasic and ON-tonic response periods (Fig 4E; Friedman ANOVA, Chi^2^ < 4.87, NS). During the OFF phase (Fig 4B), however, the RF overlap between UV and green was significantly lower than between blue and green (Fig 4E; Friedman ANOVA; Chi^2^ = 7.60, p < 0.05; Wilcoxon test, p<0.05). In blue-green narrow-band units (Fig 4C), the overlap between blue and green RFs was significantly higher than between UV and the other stimuli (Fig 4E; Friedman ANOVA, Chi^2^ = 9.58, p < 0.01; Wilcoxon test, p<0.05). The latter result was expected since these neurons presented virtually no response to UV light (Fig 4C).

Color-opponent neurons usually presented rather large RFs but sometimes with small spots of high sensitivity to the particular spectral stimuli, especially during on-phasic and off-phasic response periods (Fig 5). In both UV+/B-/G- (Fig 5A) and UV(s)/B(f)/G(f) units (Fig 5B), RFs during the on-tonic phase were composed of excitatory and inhibitory regions, predominantly for blue and green lights (Fig 5A and 5B). RFs were typically composed of excitatory and inhibitory sub-regions during the on-tonic period (Fig 5B), although the PSTH of UV(s)/B(f)/G(f) units, calculated as the average spike-rates in all RF positions (Fig 3B), did not clearly reveal inhibitory responses. RF sizes for green light were significantly larger than those for blue or UV lights (Fig 5C; Friedman ANOVA, Chi^2^ = 62.80, color effect: p < 0.0001; Wilcoxon test, green *vs* blue: p < 0.001, green *vs* UV: p < 0.0001). Furthermore, RF sizes were significantly larger for blue than UV (Wilcoxon test, UV *vs* blue: p < 0.001). The RF size measured for green and blue in the short axis (Fig 5C; Friedman ANOVA, Chi^2^ = 27.66, color effect: p < 0.0001; Wilcoxon test, green *vs* blue: NS) were significantly larger than for UV light (Wilcoxon test, green *vs* UV: p < 0.001; blue *vs* UV: p < 0.001). We found no significant differences between the RF sizes of the four different color-opponent neuron types (Table 1; GLM; type effect: F = 1.92, df = 3, NS; type x color interaction: F = 1.56, df = 6, NS).

The overlap between the large excitatory RFs recorded in the on-phasic stage did not significantly differ between pairs of stimuli in color-opponent neurons (Fig 5D; Friedman ANOVA, Chi^2^ = 3.39, NS). However, the overlap between inhibitory RFs during the on-tonic stage were significantly different for all pairs of stimuli (Fig 5D; Friedman ANOVA, Chi^2^ = 12.10; p <0.01; Wilcoxon test, p<0.05). The spatial overlap of the inhibitory responses was higher between blue and green RFs and lower between UV and green RFs (Wilcoxon test, p<0.05). Furthermore, overlap between UV and green excitatory RFs was significantly lower than between blue and green, during the off-response phase (Fig 5D; Friedman ANOVA; Chi^2^ = 8.32; Wilcoxon test, p<0.05). In line with UV *vs* blue/green spectral opponency (Fig 3), RF similarity was lower between UV and green and higher between blue and green (Fig 5D), suggesting spatial coding of chromatic information by these rather large-field neurons with their subregions of higher chromatic sensitivity.

### Spatial opponency in receptive fields

**Fig 6:**
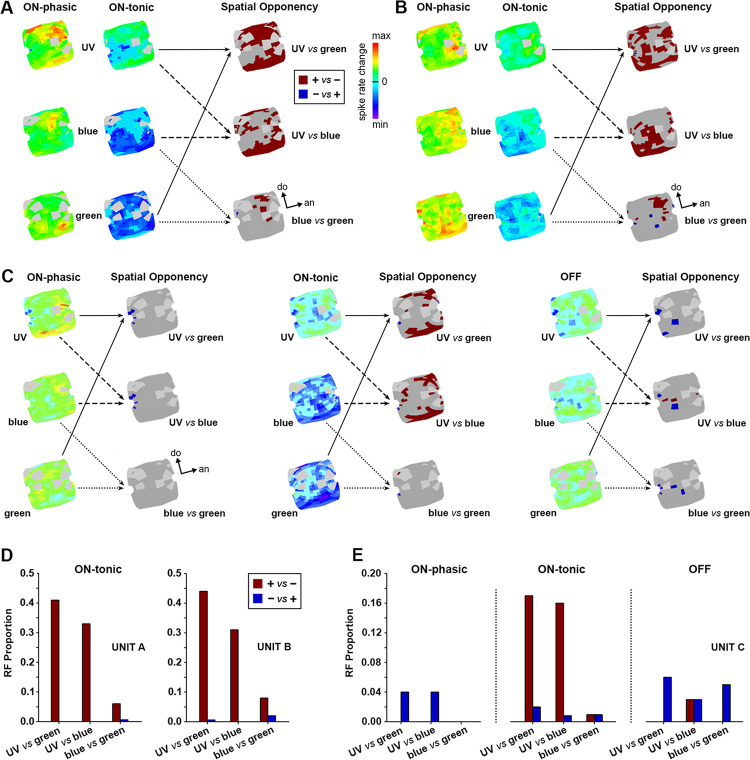
Receptive fields of some color-opponent AOT units with both double spectral and spatial opponency. Regions of the RF presenting spatial opponency during their on-tonic response phase are shown for a UV+/B-/G- unit (**A**) and a UV(s)/B(f)/G(f) unit (**B**), as indicated by dark red or dark blue on the overlapped RFs for each color pair: UV *vs* green, UV *vs* blue, blue *vs* green. Dark red indicates regions excited by the first and inhibited by the second color of each pair, whereas dark blue indicates regions inhibited by the first and excited by the second color of each pair. The units shown in (**A**) and (**B**) correspond to the ones presented in Fig 5A and 5B, respectively. They both presented large regions of their on-tonic RFs that were excited by UV and inhibited by blue or green. Some smaller RF regions were excited by blue and inhibited by green or vice-versa, during the on-tonic period (**A**-**B**). **C**: Another UV+/B-/G- unit presented RF regions with spatial opponency during all three response periods: On-phasic, on-tonic and off-phasic. As observed for the other color-opponent units (**A-B**), spatial opponency was broader during the on-tonic phase for UV *vs* blue or green (dark red). Light gray indicates regions of the RF where light stimulation failed or appeared to be unreliable. Dark gray regions of the RF did not present spatial opponency properties. **D**: Proportion of the RF presenting spectral and spatial opponency during the on-tonic response in UNIT A (**A**) and UNIT B (**B**) for each color pair. **E**: Proportion of the RF presenting spectral and spatial opponency during all response phases in UNIT C (**C**) for each color pair. Double opponency, as revealed by the simultaneous presence of red and blue bars in a same response phase, was detected during the on-tonic responses of UNITs B and C, as well as the off response phase of UNIT C. Each of the three example units (A, B and C) were recorded in a different bee.

Analyses of color-opponent RFs during the on-tonic period revealed additional spatial opponency phenomena (Figs 5A, 5B, 6A and 6B). In some of the recorded color-opponent units, certain regions of the RF were excited by one or two spectral lights and inhibited by the other (Fig 6A and 6B). These regions of spectral / spatial opponency were typically excited by UV and inhibited by blue or green, both in UV+/B-/G- (Fig 6A) and UV(s)/B(f)/G(f) neurons (Fig 6B). We also found RF regions that were excited by blue and inhibited by green, and/or vice-versa, during the on-tonic period (Fig 6A and 6B). Although spatial opponency was usually observed only during the tonic response, a few color-opponent units had spatial opponency during the on-phasic and off-phasic periods (Fig 6C). Double opponency was sometimes detected during the on-tonic (e.g. Fig 6B; blue *vs* green) or the off response phase (e.g. Fig 6C; UV *vs* blue). These latter neurons had RFs characterized by excitation to one color and inhibition to the other combined with adjacent regions of reversed color opponency (Fig 6B and 6C). For example, the unit shown in Fig 6B presented double opponency for blue and green during the on-tonic response phase (Fig 6D, UNIT B). Similar response properties were detected for the unit shown in Fig 6C, which also presented double opponency for UV and blue during the OFF response phase (Fig 6E, UNIT C). RF regions presenting UV *vs* green or UV *vs* blue spatial opponency were typically larger than the ones presenting blue *vs* green spatial opponency during the on-tonic phase (Fig 6D and 6E). Units presenting spatial-opponency along all response phases (*e*.*g*. Fig 6C) had larger proportions of opponent RF regions during the on-tonic than the on-phasic or off-phasic response periods (Fig 6E).

### Color combination and opponency phenomena

To better evaluate and uncover hidden color-opponency phenomena in AOT neurons, we recorded from 21 units using a stimulation protocol that presented combinations of distinct spectral lights (UV-blue, UV-green, blue-green and UV-blue-green).

### Simultaneous presentation of UV, blue and green

**Fig 7:**
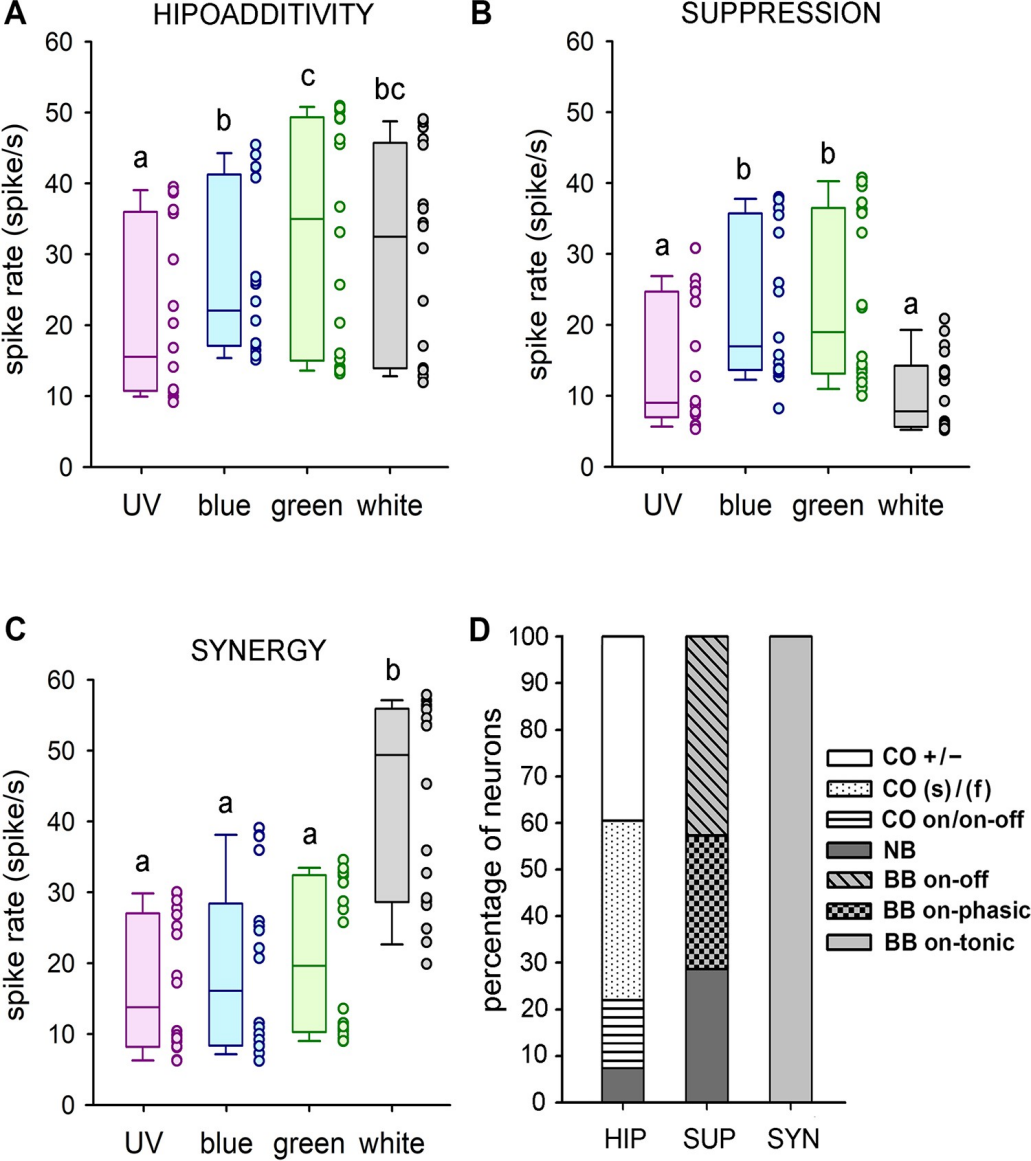
Responses to a mixture of UV, blue, green light (bee´s white) in comparison to the single spectral components reveal hipoadditive (A), suppressive (B) or synergic (C) combinatory effects. Box plots represent the spike rates of each example neuron (A, B and C) from values recorded in three presentations of each stimulus over six distinct RF coordinates. Each of the three example units (A, B and C) were recorded in a different bee. The horizontal line within the box shows the median. The boundaries of the box indicate the 25th and 75th percentile. Whiskers indicate the minimum and maximum values. The dots at the right of each box show data dispersion (N = 18 presentations per stimulus). Different letters indicate significant differences (p < 0.05) in Wilcoxon matched-pairs tests. **D**: Types of neurons displaying each combinatory effect.

**Fig 8:**
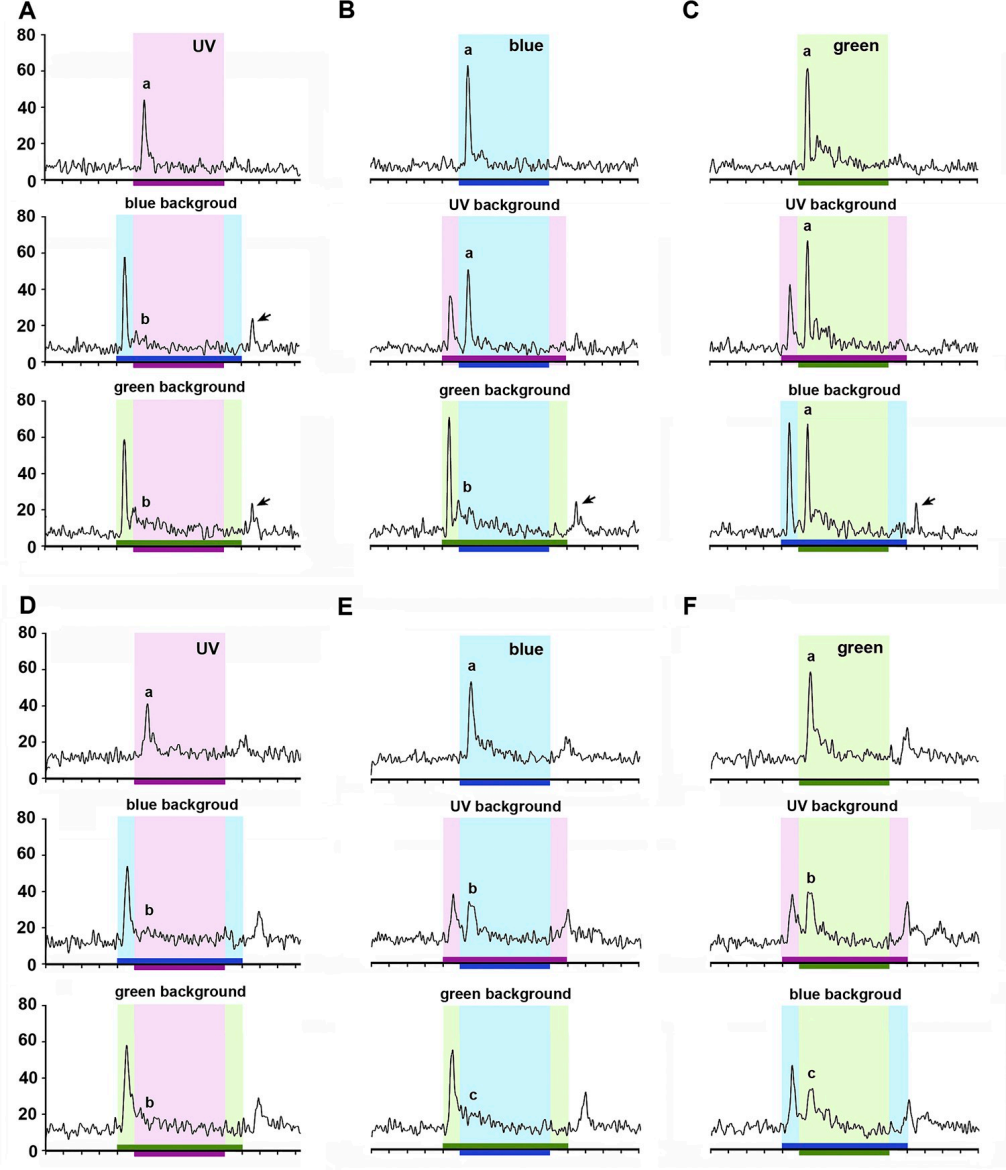
Broad-band (BB) neurons in response to chromatic combinations. **A-C:** Responses of a BB ON-phasic neuron to UV (**A**), blue (**B**) and green (**C**) stimulus over distinct backgrounds. Arrows indicate OFF-responses that emerged after chromatic combination. **D-F**: Responses of a BB ON-OFF neuron to UV (**A**), blue (**B**) and green (**C**) stimulus over distinct backgrounds. Bins on the abscissa indicate 100ms time intervals. Horizontal bars indicate background (700ms) and stimulus (500ms) presentation. Different letters indicate significant difference between spike rate during the onset of each spectral light presented alone or in the presence of two distinct spectral backgrounds (Friedman ANOVA; A-B and D-F: Chi^2^ > 31.71, p < 0.01; Wilcoxon test, A-F: p < 0.02 for all significant comparisons).

Simultaneous presentation of UV, blue and green revealed that most AOT units present some level of spectral-opponency, as indicated by reduced additivity (hipoadditive, n = 13; Fig 7A) or suppressive responses (n = 7; Fig 7B) to color combinations. Only one unit out of 21 presented additive (synergic) responses to the color mixture (Fig 7C). Green light was the spectral component that induced highest excitation in most recorded units (n = 14; e.g. Fig 7A; Friedman ANOVA, Chi^2^ = 13.60, p < 0.02; Wilcoxon test, green *vs* UV or blue: p<0.03), but in some cases blue also produced equal excitation (n = 7; e.g. Fig 7B; Friedman ANOVA, Chi^2^ = 49.74, p < 0.001; Wilcoxon test, green *vs* blue: NS). Response magnitudes to the UV-blue-green mixtures (a mixture that will be close to “bee’s white”) were not higher than that to the most effective light (hipoadditivity; e.g. Fig 7A; Wilcoxon test, white *vs* green: NS) in five *UV+ / B- / G-* (e.g. Fig 3A), five *UV(s)/B(f)/G(f)* (e.g. Fig 3B) and two *UV(on) / B(on-off) / G(on-off)* color-opponent neurons (e.g. Fig 3D), as well as one *BG+* narrow-band neuron (e.g. Figs 2D and 7D: *HIP*). Lower activation to the UV-blue-green mixture than to the strongest component (suppression; Fig 7B; Wilcoxon test, white *vs* blue or green: p < 0.001) was observed in two *UV+/B+/G+* on-phasic broad-band neurons (e.g. Fig 2B), three *UV+/B+/G+* on-off broad-band neurons (e.g. Fig 2C) and two *BG+* narrow-band neurons (e.g. Figs 2D and 7D: *SUP*). One single unit was found with higher excitation to the UV-blue-green mixture than to the strongest component (synergy; Fig 8C; Friedman ANOVA, Chi^2^ = 29.76, p < 0.01; Wilcoxon test, white *vs* UV, blue or green: p<0.005) and was classified based on its responses to each spectral component as a *UV+/B+/G+* ON-tonic broad-band neuron (e.g. Figs 2A and 7D: *SYN*).

These results demonstrate that even some units classified as broad-band or narrow-band according to their responses to UV, blue and green, are probably connected to color-opponent circuits that promote hipoadditivity or suppression (Fig 7D) in response to the bee’s white light (trichromatic mixture).

### Background light and color addition effect

**Fig 9:**
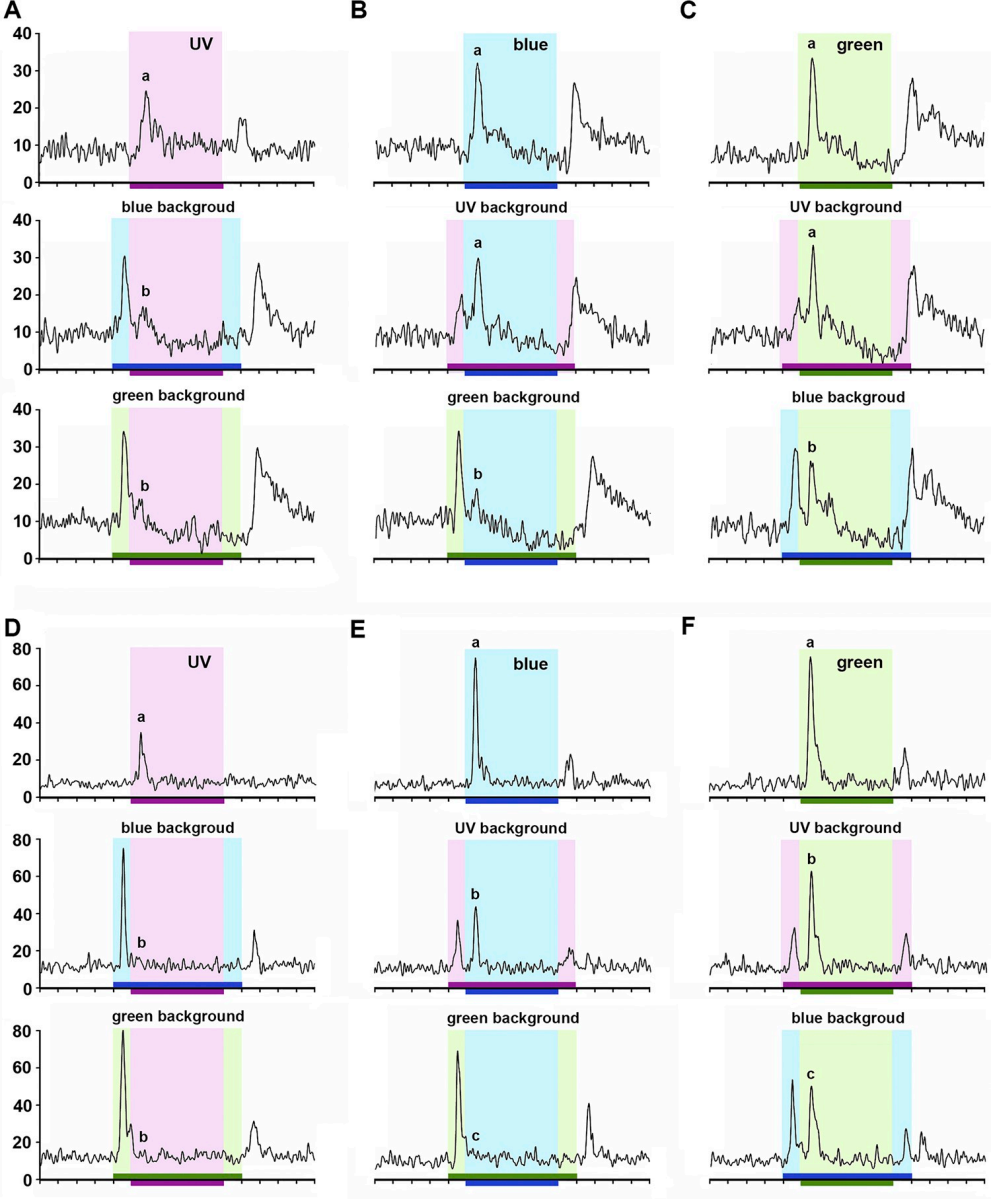
Color-opponent neurons in response to chromatic combinations. **A-C:** Responses of a UV+/B-/G- neuron to UV (**A**), blue (**B**) and green (**C**) stimulus over distinct backgrounds. **D-F:** Responses of a UV(on)/B(on-off)/G(on-off) neuron to UV (**A**), blue (**B**) and green (**C**) stimulus over distinct backgrounds. Bins on the abscissa indicate 100ms time intervals. Horizontal bars indicate background (700ms) and stimulus (500ms) presentation. Different letters indicate significant differences between spike rates during the onset of each spectral light presented alone or in the presence of two distinct spectral backgrounds (Friedman ANOVA; A-F: Chi^2^ > 25.56, p < 0.01; Wilcoxon test, A-F: p < 0.03 for all significant comparisons).

We analyzed how responses to a specific spectral light changed when presented in addition to another background light, for example UV on a blue or green background, blue on a UV or green background, green on a UV or blue background (Figs 8 and 9). Curiously, we found that in both broad-band (Fig 8) and color-opponent units (Fig 9) responses to a given color were often reduced or even extinguished when a background color was present. Apart from one unit that presented additive responses to some color combinations, all other 20 neurons recorded shared the following on-phasic response properties (Figs 8 and 9). In the presence of blue or green background light, responses to UV were completely (Figs 8A, 8D and 9D) or almost completely inhibited (Fig 9A). Thus green and blue “dominated” over UV. In the presence of UV background, responses to blue were unaltered (Figs 8B and 9B) or partially inhibited (Figs 8E and 9E). In the presence of green background light, responses to blue were completely (Figs 8B, 8E and 9E) or partially inhibited (Fig 8B). Thus green “dominated” blue; blue “dominated” UV. In the presence of UV or blue light, responses to green were not affected (Fig 8C) or partially inhibited (Figs 8F, 9C and 9F). Thus green “dominated” blue and UV. Overall, dominance of longer wavelengths over shorter ones seems to be a common physiological principle of the on-phasic responses in the AOT circuitry.

Complete spectral dominance was typically observed in broad-band ON-phasic neurons (Fig 8A–8C). After total inhibition of responses to a shorter wavelength by a longer-wavelength background, off-responses often followed the offset of the background light (Fig 8C, arrows). Broad-band ON-OFF neurons usually presented responses with partial wavelength dominance (Fig 8D–8F). Whereas blue and green backgrounds equally suppressed responses to UV (Fig 8D), inhibition of blue was stronger by green than UV background (Fig 8E) and inhibition of green was stronger by blue than UV background (Fig 8F).

We found in these color addition experiments a consistent difference between responses in UV+/B-/G- (Fig 9A–9C) and UV(on)/B(on-off)/G(on-off) color-opponent neurons (Fig 9D–9F). Whereas UV background did not affect responses to blue or green in the former (Fig 9B and 9C), it provoked partial inhibition in the latter neurons (Fig 9E and 9F). Some UV(s)/B(f)/G(f) color-opponent neurons responded like UV+/B-/G- units (Fig 9B and 9C) while others were similar to UV(on)/B(on-off)/G(on-off) units (Fig 9E and 9F). Blue-green narrow-band neurons typically presented total inhibition of blue on-phasic response by green background (like Figs 8E and 9E) and partial inhibition of green on-phasic response by blue background (like Fig 9F).

## Discussion

**Fig 10:**
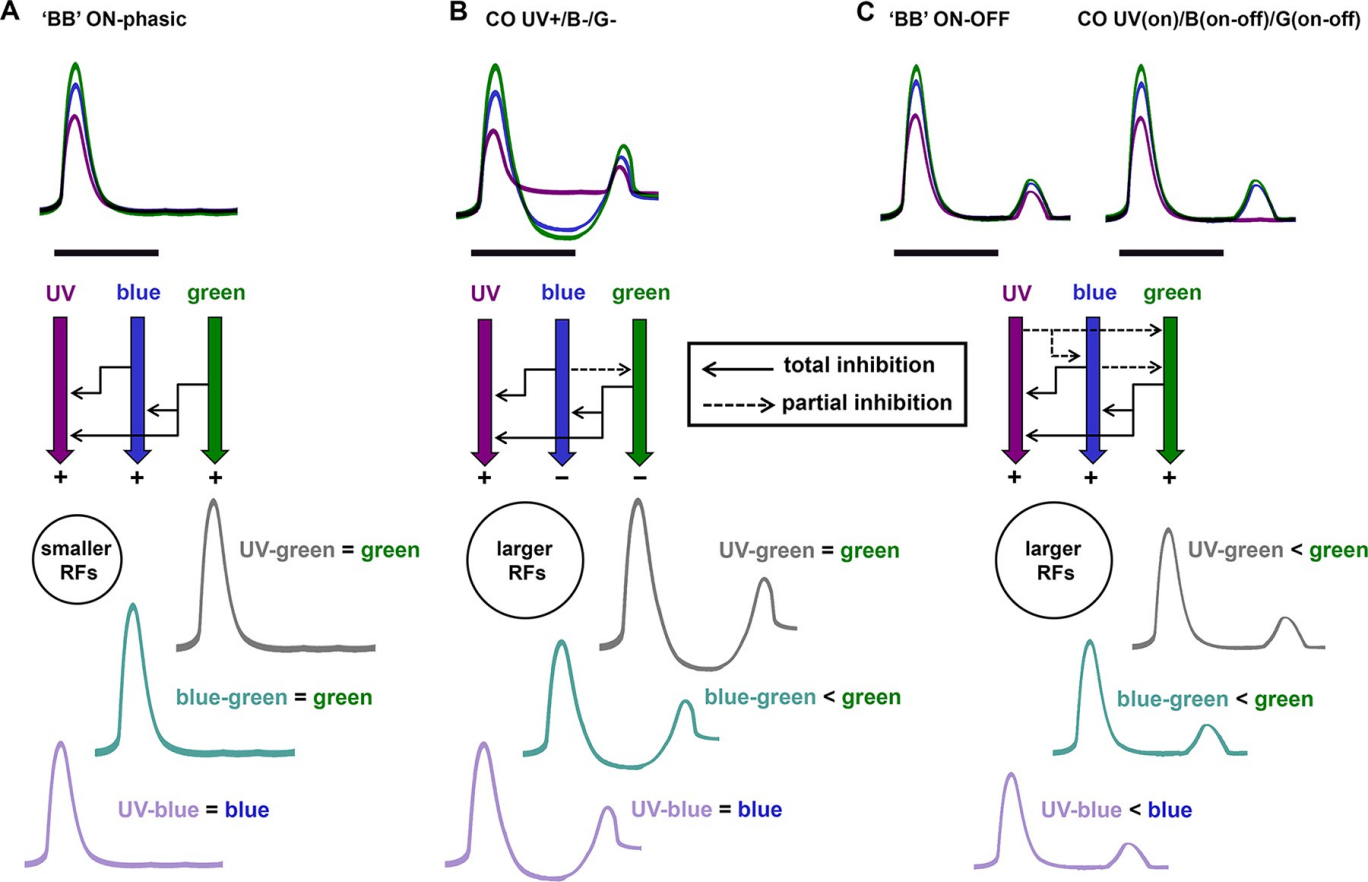
Three types of spectral dominance recorded in AOT neurons of the honeybee, and their associated inhibitory pathways, as revealed by responses to chromatic combinations. A) Broad-band (BB) ON-phasic neurons present similar excitatory responses to each spectral stimulus (upper curves). However, responses to chromatic mixtures reveal complete domination of longer wavelengths over shorter wavelengths (lower curves), suggesting inhibitory pathways (black arrows) that produce this suppressive phenomena. These neurons usually present smaller receptive fields (RFs) than BB ON-OFF or any color-opponent (CO) neuron type. B) CO UV+/B-/G- neurons respond with tonic excitation to UV and tonic inhibition to blue or green (upper curves). While a background of longer wavelength (e.g. green) inhibits (solid arrows) responses to any shorter wavelength (e.g. UV) added to the mixture (lower curves), a blue background also partially inhibits responses to green (dashed arrow). C) BB ON-OFF and CO UV(on)/B(on-off)/G(on-off) neurons while different in their responses to individual spectral stimuli (upper curves) presented very similar responses to chromatic mixtures (lower curves), with total inhibition of shorter wavelengths by longer wavelengths (solid arrows) and partial inhibition of longer wavelengths by a background of shorter wavelength. Therefore, ‘BB’ neurons as the ones illustrated in (A) and (C) are also connected to color-opponent circuits. Neuronal categories presenting OFF responses to single spectral stimuli and partial inhibition (dashed arrows) of shorter over longer wavelengths (B-C) have larger RFs than ON-phasic neurons (A).

We recorded the activity of 72 units in the anterior optic tract (AOT) of the honeybee using UV, blue and green light stimuli presented in 266 positions of the visual field and found that the majority of these units present combined chromatic-spatial processing properties. Quantifying and comparing neuronal activity to each of these spectral stimuli, we found eight different neuron categories in terms of their spectral and temporal response properties (Table 1). Color-opponent AOT neurons, the most abundant neural category in the AOT, presented large receptive fields and activity patterns that were typically antagonistic between UV and blue or green, particularly during the on-tonic response phase (Fig 5). Receptive field shapes and activity patterns of these color processing neurons were more similar between blue and green, than between UV and blue or green (Fig 5C and 5D). We also identified intricate spatial antagonism and double spectral opponency in some receptive fields of color-opponent units (Fig 6). Furthermore, stimulation protocols with different color combinations applied to 21 AOT units allowed us to uncover other levels of spectral antagonism and hidden inhibitory inputs, even in some units that were formerly classified as broad-band neurons based in their responses to single spectral lights (Figs 7–9). While belonging to diverse neural categories in terms of spectral and spatiotemporal properties, most AOT units recorded shared a similar color coding principle in which long wavelengths partially or totally dominate short wavelengths when a pair of distinct lights are combined (Figs 8–10). Taken together, these properties resemble in general those found in some other animal species. However, concentric single or double opponent receptive fields as they are found in e.g. mammalian visual interneurons are not found [52]. This characteristic difference together with the usually large receptive fields and their subfields indicate more specified neural coding strategies of the AOT neurons possibly adapted to subfunctions of visual perception and visual control.

### Anatomical categories of AOTU neurons

The anterior optic tubercle (AOTU) is the most prominent optic glomerulus in the insect anterior lateral protocerebrum, but little is so far known about the function of this brain region in any insect. While traditionally studied in locusts and butterflies as a brain region involved in processing polarized skylight [53], previous works [40,42,43] and this study indicate an involvement of the bee AOTU in spatial and chromatic processing (see next discussion section). Recently, the AOTU has been shown to participate in figure-ground discrimination in *Drosophila* [54], to be volumetrically plastic in *Camponotus* ants exposed to a color memory task [55], and to modulate visual social processing through facial recognition in the wasp *Polistes fuscatus* [56].

The honeybee AOTU receives visual input from the medulla and lobula via the AOT, a massive bundle of ~ 50μm diameter, which projects ventro-anteriorly from the proximal lobula and enters the AOTu laterally [41]. Anatomical analyses of AOT neurons in bees revealed a mixed population of ~ 230 neurons, comprising at least three distinct categories: transmedulary (columnar) small-field neurons; lobula (columnar) small-field neurons from distal layers 1 to 4; lobula tangential neurons from proximal layers 5 and 6 [41,44]. These neurons comprise two parallel visual pathways providing segregated input from the dorsal and ventral eye regions to distinct AOTu compartments. While the AOTU lower unit complex (LUC) receives information exclusively from the dorsal medulla, neural circuits of the upper unit (UU; previously called the major unit—MU) receives information from the dorsal and ventral parts of the medulla and lobula in a segregated manner [41,45].

The LUC of the AOTU receives visual input from the dorsal rim area (DRA) of the honeybee compound eyes via the dorsal medulla, and sends projections to the synaptic microglomerular complexes of the lateral (LBU) and medial bulbs (MBU) in the central brain region [46,57,58]. As described in locusts [59], crickets [60], butterflies [61] and *Drosophila* [62,63], neurons of this neural pathway compose the so called “sky compass network” that processes the pattern of polarized light and the sun azimuth in the sky [53]. The large majority of neurophysiological works performed so far in the AOTU were specifically focused in polarized light processing involving the LUC [53,63]. In locusts, intracellular recordings of some AOT neurons connecting to the LUC indicated complex receptive field properties with spectrally and spatially opponent subfields [59,64,65]. These authors used unpolarized green light stimulus that mimics the sun spot in the UV/blue sky. They suggest these color-opponent neurons of the locust LUC could be involved in the processing of solar *vs* anti-solar sky gradients. While neurons of the LUC have been so far the most studied, the majority of AOT neurons send projections to the honeybee AOTU-UU [41], but the physiological properties of these input neurons are poorly described in any insect.

### Spectral response properties

In the present study we recorded the activity of multiple AOT neurons using extracellular electrophysiology with copper electrodes. Although the electrode tips were covered with dextran-coupled dye to allow further appraisal of electrode positions within the prominent AOT, the morphology of single units recorded could not be elucidated from this fluorescent staining. Yet, our work represents the first description of the receptive-field structure and spectral properties of distinct categories of AOT neurons in honeybees. Few studies have so far described the physiology of neurons connecting to the AOTU of bees. A single neuron connecting the honeybee AOTU-UU to a contralateral protocerebral region was found to be binocularly sensitive to stationary white light and to moving stimuli, with no directional selectivity [33]. Some input neurons from the lobula to the AOTU-UU and output neurons from the AOTU-UU to the posterior protocerebrum of bumblebees were found to be mainly color sensitive, with no sensitivity to directional motion cues [40,42]. None of these intracellular electrophysiological studies, however, described in detail the shape and properties of the receptive fields of the AOTU neurons recorded.

Large receptive fields and spectral opponency were the predominant patterns of AOT neurons recorded in the present study. It is then possible that we recorded a majority of lobula neurons connecting the proximal lobular layers 5 and 6 to the AOTU, because they presented wider dendritic arborizations than the small-field columnar neurons connecting lobula distal layers 1 to 4 or the medulla to the AOTU [41]. Moreover, layers 5–6 lobula neurons were shown to present a majority of narrow-band or color-opponent neurons in bumblebees, while the majority of layers 1–4 lobula neurons were chromatically insensitive [40]. Thus the broad-band on-phasic AOT units presenting smaller receptive fields recorded in our work may originate in the lobula distal layers 1 to 4. Neurons with smaller receptive fields may also be transmedulary columnar neurons connecting the medulla directly to the AOTU [41,44]. No study has so far described the activity of transmedulary neurons connecting directly to the AOTU of bees, but some transmedulary neurons connecting to the lobula were found to have narrow-band or broad-band responses patterns in honeybees and the bumblebees [31,42].

In terms of neural activity to single spectral stimuli, response properties of all 34 color-opponent AOT units were similar for green and blue, while opponent for UV (eg. UV+/B-/G-). Interestingly, this was also the predominant color opponency pattern identified in the optic lobes of honeybees, with a great majority of UV+/B-/G- and rare UV-/B+/G+ units recorded [28]. Whereas no UV inhibited neuron was recorded in this study, we identified UV+/B-/G- as the main color-opponent category in the AOT of honeybees, as previously described in the proximal layers of the medulla [28]. While at least seven distinct color-opponency categories involving excitation versus inhibition were already described in the bee optic lobes [28,31,37,40,42], we only recorded UV+/B-/G- neurons in the AOT of honeybees. We also found three other types of UV versus blue-green opponency, in which responses to UV differed from blue and green in their temporal properties: 1-slower adaptation, 2-faster adaptation, 3-no OFF-component (Fig 3).

Despite the apparent similarity of responses to blue and green, color combination protocols with one color presented as background of another color revealed hidden opponent effects between blue and green in all AOT color-opponent neurons (Fig 9). Responses of AOT neurons to color combinations also revealed that even units initially classified as broad-band on-phasic or on-off phasic based in their responses to single spectral lights are actually connected to color-opponent circuits that promote partial or total suppressive effects (Fig 8). A main principle appears in almost all the units recorded using color combination protocols (n = 20 from 21): a background of a longer wavelength drastically inhibits responses to any shorter wavelength e.g. green suppresses UV or blue, blue suppresses UV (Figs 8 and 9). On the other hand, a background of a shorter wavelength had no effect on the response to longer wavelengths in some neural types, while partially inhibits the response to longer wavelengths in other units (Figs 8 and 9). Since neurotransmission involving the AOT circuitry [41,45] is yet poorly described in bees, further studies will have to include pharmacological approaches to elucidate the sources of excitation and inhibition found in our study.

Fig 10 summarizes the three categories of spectral dominance identified in honeybee AOT neurons and associated inhibitory pathways that were apparently hidden when responses to single spectral stimuli were recorded in each category. All units classified as broad-band ON-phasic, although displaying similar responses to UV, blue or green alone, presented total inhibition of longer wavelengths over short wavelengths in responses to color combination (Fig 10A). This same pattern of inhibition was also observed in color-opponent and broad-band ON-OFF neurons (Fig 10B and 10C). However, UV+/B-/G- color-opponent neurons typically presented partial inhibition of responses to green when blue was first presented as a background color (Fig 10B). Surprisingly, all the other categories of color-opponent neurons, as well as broad-band ON-OFF neurons, presented not only partial inhibition of responses to green by a blue background, but also partial inhibition of responses to blue and green by a UV background (Fig 10C). Taken together these results reveal that the large majority of neurons initially classified as broad-band neurons based in their responses to single UV, blue and green, are actually connected to consistent color-opponent circuits. Curiously, neuronal types presenting OFF responses to single spectral stimuli and partial inhibitory effects of shorter wavelengths over longer ones (Fig 10B and 10C) presented larger receptive fields than ON-phasic neurons (Fig 10A). Both the larger receptive fields and the OFF-responses may be related to a higher amount of lateral inhibition in the circuitry of those neurons (Fig 10).

Only one broad-band ON-tonic unit did not respond with suppressive effects, but instead presented synergic responses to color combinations (Fig 7). This AOT neuron category thus appears to involve processing of light intensity rather than chromatic information, as compared to the large majority of the AOT units recorded in the present study. These neurons usually presented smaller receptive fields than broad-band ON-OFF or color-opponent neurons. We cannot exclude the possibility that the size and/or anatomy of AOT large-field color processing neurons favored a higher sampling rate of those neurons in our electrophysiological approach. Since we used gold plating of the very thin cooper electrodes, providing lower impedances, we should also record small neurons with thinner axons and not only large and thick neurons. Complementary studies using intracellular electrophysiology are desirable for better characterizing the relation between neuron anatomy and activity pattern, as well as the specific spike type properties (e.g. amplitude, slope and decay time) in distinct AOT neurons of the bee brain.

Calcium imaging of intertubercle neurons connecting the AOTU of the two honeybee brain hemispheres revealed that the activity patterns evoked by dorsal and ventral eye stimulation were clearly segregated into different subcompartments of the upper unit—UU [41]. These same intertubercle circuits were found to present spatiotemporal color coding properties, with distinct response patterns to UV, blue and green, as well as clear inhibitory phenomena in responses to blue-green color combinations [43]. Thus color-opponent processing here described at the input (AOT) level of the AOTU circuitry is also retained at the output level to the contralateral AOTU, and other deeper protocerebral regions [40,42,43]. On-phasic spike-rate frequencies of the AOT neurons recorded here were typically higher for green and blue than for UV presented in a same absolute intensity. In agreement, the amplitude of calcium signals and the area of activation (in pixels) recorded in AOTU intertubercle neurons was consistently higher for green, followed by blue and UV (green>blue>UV) presented at same absolute intensity [40,42,43]. As well as in the large majority of AOT neurons here recorded, responses of intertubercle output circuits to distinct combinations of blue and green were always equal to (hipoadditivity) or weaker (suppression) than responses to the green component of the chromatic mixture [40,42,43].

### The role of AOT neurons in color vision

Floral color patterns are well discriminated by honeybees if they exceed the spatial resolution of color vision (>15° visual angle, [66–68]). The neural processes of color pattern recognition are practically unknown because of the lack of recordings of the receptive fields of higher order visual interneurons of the bee brain. From an ecological point of view, one might expect a division between a floral color pattern analyzing neural system and one that analyses the celestial compass. In the first case, floral color patterns are fine grain on a green or grey (bee white) background, in the second case a spot like green target (sun) on a broad gradient ranging from UV to blue background (sky).

Here we recorded from neurons of the anterior optic tract (AOT) that sends massive input from the visual ganglia to the AOTU [41]. As mentioned before, some few neurons of this prominent neural tract sends input exclusively from the dorsal region of the eye and medulla to the LUC [41,45], a sub-compartment specialized in polarized light and celestial compass processing in different insects. Although there is no physiological study performed so far in the LUC of the honeybee ATOU, the neural organization and connectivity of this small subunit in bees [41,45,46] highly resembles those of other insects in which the properties of LUC neurons in processing sky cues have been described [53,63]. Most of the numerous AOT neurons provide information from regions of the whole receptive field of the bee compound eye to the UU, a major AOTU unit which is thus expected to be in the neural stream more closely related to floral color pattern recognition.

This expectation is confirmed by the spectral properties and receptive fields (RFs) of the diverse AOT neurons as described here: (1) The RFs were not limited to the upper/dorsal part of the eye (Figs 4 and 5); (2) The complex temporal dynamic of the RF structure suggests a role in fast scanning behavior during the detection of close colored objects rather than the imaging of a large field of chromatic gradient (Fig 6); (3) UV and blue stimuli on a green background as well as UV stimuli on a blue background lead to contrasting responses (Figs 8–10). Thus we conclude that most of the AOT neurons recorded in our study, which probably arborize in the UU, are involved in the processing of multitudes of close and localized colored objects (flowers) rather than the chromatic analysis of the sky in bees.

A surprising result of our study is the complexity of color opponent and double color/spatial opponent units. In this sense, color coding in higher order neurons in the bee brain differs from color coding in the vertebrate brain that is characterized by units with ring-like concentric single or double opponency and complex double opponency [69,70].

The temporal dynamic of the AOT units described here in the double stimulus experiments (Figs 8–10) is unique, not seen in color coding neurons in the vertebrate brain and so far unknown from color coding neurons in invertebrate brains. The response patterns appear as highly diverse and specific to the particular combinations and sequences of the dual spectral stimulations. However, a general rule appears to apply to the different effects of longer wave background vs to shorter wave background: green background drastically suppresses responses to UV and blue local stimuli, blue background suppresses local UV stimuli (Figs 8–10).

It is tempting to relate these properties to color constancy phenomena since natural objects like flowers are illuminated predominantly by longer wavelengths. A bee approaching a flower will scan the colored objects against a background that is illuminated by the same light as the object. The excitations in the three spectral receptor pathways will change when the chromaticity of the illumination changes. Neural processing underlying color constancy has been related to the calculation of proportions of excitations between object and background in the three spectral pathways (retinex theory, [71]). The changes between object and background are simulated by our double stimulus experiments. Longer wave opponency to shorter wave local cues may reflect a proportional coding rather than an absolute coding of stimulation. Further experiments would have to test whether the responses of AOT neurons to the chromatic properties of the object code the proportion of the illuminations rather than the absolute values, as previously shown in behavioral experiments [9,20], properties known from complex double opponent neurons in the cortex of monkeys [72].

## Abbreviations

AL: antennal lobe
ANOVA: analysis of variance
AOT: anterior optic tract
AOTU: anterior optic tubercle
CA: calix of the mushroom body
CB: central body
DRA: dorsal rim area
GLM: generalized linear model
LA: lamina
LBU: : lateral bulb
LED: light emitting diode
LO: lobula
LUC: lower unit complex
LX: lateral complex
MBU: medial bulbs
ME: medulla
MU: major unit
PCA: principal component analysis
PSTH: peristimulus time histogram
PWM: pulse width modulation
RF: receptive field
SD: standard deviation
TALT: tubercle accessory-lobe tract
UU: upper unit
UV: ultraviolet
vITT: ventral intertubercle tract
